# Whole-Genome Sequences of *Staphylococcus haemolyticus* Isolated from Infected Eyes and Healthy Conjunctiva in Bhubaneswar, India

**DOI:** 10.1128/genomeA.00099-16

**Published:** 2016-03-17

**Authors:** Sasmita Panda, Durg V. Singh

**Affiliations:** Infectious Disease Biology, Institute of Life Sciences, Bhubaneswar, India

## Abstract

*Staphylococcus haemolyticus*, an opportunistic pathogen, is known to exhibit multidrug resistance and produce biofilm. We sequenced the genome of four multidrug resistant, biofilm forming isolates from infected eyes and asymptomatic healthy conjunctiva.

## GENOME ANNOUNCEMENT

*Staphylococcus haemolyticus*, known to cause infection in immunocompromised hosts and catheter-associated infections, ranks second among coagulase-negative staphylococci (CoNS) after *S. epidermidis* in the frequency of isolation from blood cultures (1). *S. haemolyticus* is also known to cause septicemia, peritonitis, otitis, and urinary tract infections. Ocular infections caused by *S. haemolyticus* have also been reported (2). This organism is known to exhibit resistance to several antibiotics including heteroresistance to glycopeptides (3, 4). The whole-genome sequence of *S. haemolyticus* JCSC1435 reported by Takeuchi and coworkers (5) revealed the presence of insertion sequences in the genome that could encourage frequent genomic rearrangements.

We have a cohort of 18 isolates of *S. haemolyticus*, of which, eleven were from infected eyes and seven from asymptomatic healthy conjunctiva. Fifteen out of 18 isolates, irrespective of sources of isolation, were multidrug resistant, and all isolates had the ability to form a biofilm. We selected four out of the 18 isolates, two from infected eyes and two from healthy conjunctiva. These strains showed different pulsotypes indicating diversity in the genomic content among them (unpublished data). Moreover, *S. haemolyticus* isolates SH747 (infected eye), SH1574 (infected eye), SHN36 (healthy conjunctiva), and SHN65 (healthy conjunctiva) belonged to sequence types (STs) 22, 1, 27, and 21, respectively (unpublished data). This study reports the whole-genome sequences of the four isolates, which are prerequisites for understanding the molecular mechanisms of antibiotic resistance, virulence, and biofilm formation in *S. haemolyticus*, with particular attention on their source of isolation.

Genomic DNA from *S. haemolyticus* was extracted by the phenol-chloroform extraction method. Whole-genome sequencing was performed using the 454 Roche and Illumina sequencing platforms at NxGenBio Life Sciences, Delhi. The reads obtained were quality filtered using the FastQC toolkit. Sequence data of all the isolates were *de novo* assembled with the help of Newbler *de novo* assembler. The resulting assemblies generated 278, 334, 179, and 339 contigs representing the genomes of *S. haemolyticus* isolates SH747, SH1574, SHN36, and SHN65, with an average sequencing coverage of 481×, 537×, 456×, and 560×, respectively. *De novo* assembled sequences were further annotated by submitting the sequences to Rapid Annotation using Subsystem Technology (RAST) (6).

The chromosome sizes of the four isolates of *S. haemolyticus* are 2.8 Mb (SH747), 2.6 Mb (SH1574), 2.6 Mb (SHN36), and 2.6 Mb (SHN65), respectively. The G+C content of the strains is within 32.7 to 32.9%. After annotation, it was evident that the isolates SH747, SH1574, SHN36, and SHN65 contained 1,142, 1,100, 1,082, and 1,062 protein coding genes, and 19, 19, 19, 20 RNAs, respectively. The genome analysis showed a relatively high number of coding sequences (CDs) for the amino acid, carbohydrate, protein, RNA, and DNA metabolism in all the four isolates. The availability of these sequences will facilitate the genomic comparison of isolates from infected and healthy conjunctiva.

### Nucleotide sequence accession numbers.

The genome sequences of *S. haemolyticus* isolates SH747, SH1574, SHN36, and SHN65 have been deposited in DDBJ/EMBL/GenBank under accession numbers LRHM00000000, LRBM00000000, LRBN00000000, and LRHO00000000, respectively.
